# Investigation of lncRNA-mRNA co-expression network in ETV6-RUNX1-positive pediatric B-cell acute lymphoblastic leukemia

**DOI:** 10.1371/journal.pone.0253012

**Published:** 2021-06-08

**Authors:** Weijuan Yu, Weihua Wang, Xiumei Yu

**Affiliations:** Department of Hematology Laboratory, Yantai Yuhuangding Hospital, Yantai, P.R. China; European Institute of Oncology, ITALY

## Abstract

ETV6/RUNX1 gene fusion is the most common chromosomal translocation abnormality occurred in pediatric B-cell acute lymphoblastic leukemia (B-ALL). Compared with ETV6-RUNX1-negative patients, ETV6-RUNX1-positive patients possess more improved treatment strategies but higher risk to relapse. In this research, the potential gene interaction networks were constructed intending for elucidating the pathogenesis of B-ALL. We performed the weighted gene co-expression network analysis (WGCNA) to assess the involvement of lncRNA-mRNA pairs in B-ALL patients consisting of 24 ETV6-RUNX1-positive patients and 18 ETV6-RUNX1-negative patients and found a module that was significantly associated with positive/negative trait. Gene Ontology analysis showed that mRNAs in this module were enriched in the positive regulation of MAPK cascade, positive regulation of JNK cascade, and myeloid cell differentiation pathway. To further investigate the relationship between lncRNAs and mRNAs in this significant module, we constructed the lncRNA-mRNA co-expression network. 3 lncRNAs (RP11-170J3.2, RP11-135F9.1 and RP1-151B14.9) were found at the core of the lncRNA-mRNA co-expression network, which had the most co-expression connections with mRNAs. And several related mRNAs (ACTN1, TNFRSF21 and NLRP3) had a significant correlation with the patient survival prediction. Our findings may explicate the pathogenesis of B-ALL, and the disease-associated genes could provide clues to find novel biomarkers for prognosis.

## Introduction

Acute lymphoblastic leukemia (ALL), a type of blood cancer, is the most common malignancy diagnosed in children worldwide [1], with about 90% 5-year survival rate in children, and 75–85% in adolescents and young adults [2–4]. The outcomes are worse in older adults, with an overall survival rate of 35–55% in middle-aged people and below 30% in people over 60 [5,6]. The hallmark of ALL is driven by a spectrum of genetic aberrations including mutations, chromosome translocations and aneuploidy in genes involved in differentiation and proliferation of B-lineage or T-lineage lymphoid precursor cells [7]. B-cell acute myeloid leukemia (B-ALL) accounts for 85% of ALL cases [8]. The reciprocal translocation t(12;21) (p13;q22)[ETV6/RUNX1] is the most frequent chromosomal rearrangement in B-ALL with an incidence of approximately 25% [9]. This rearrangement is associated with a favorable clinical outcome under present treatment protocol, such as intensive chemotherapy or allogeneic hematopoietic cell transplantation [10], but up to 20% of ETV6-RUNX1-positive pediatric B-ALL patients have a recurrence and still succumb to their disease [11].

Long non-coding RNAs (lncRNAs) are dysregulated in cancer, conferring the cancer cell capacities for tumor initiation, growth, and metastasis, and served as a promising target for cancer diagnosis and therapy [12]. LncRNAs are transcripts longer than 200 nucleotides in length, and have a complex secondary and tertiary structure. The expression of lncRNAs is highly spatially and temporally restricted during proliferation, differentiation, and cell death [13]. Moreover, lncRNAs locate within intergenic stretches or overlapping antisense transcripts of protein-coding genes and function as important regulators of gene expression showing cell-specific expression patterns and subcellular localization [14]. Based on the function, they are classified as guide, decoy, signaling, scaffold, or enhancer lncRNAs [15]. LncRNAs play relevant roles in the development of cancer, including leukemia. There have identified a number of lncRNAs association with several subtypes of leukemia, such as MEG3, IRAIN, and UCA1 related to acute myeloid leukemia (AML) and ANRIL, LUNAR1, in ALL [16]. Recently research identified lncRNA TCL6 was strongly related to ETV6-RUNX1-positive pediatric B-ALL, and lower TCL6 expression level may be connected with poor disease-free survival [17]. The investigation of the aberrant expressed lncRNAs could complement current cytogenetic and molecular biological analyses applied in the routine diagnosis and treatment of diseases for a better management of patients.

Several studies have investigated expression profile by employing Weighted Gene Co-expression Network Analysis (WGCNA) approach to identify prognostic genes in leukemia [18,19]. The co-expression network analysis clusters highly synergistic changed lncRNA and mRNA into modules, in which embrace the genes with similar expression patterns. The distinct advantage of WGCNA is that it can find co-expressed genes by calculating gene connectivity, and can identify candidate biomarkers from a large number of gene sets rather than a small number of differentially expressed gene sets. It providing insights into hub genes that may be responsible for phenotypic traits in various diseases [20–24].

To identify prognosis-related molecules and the potential recurrence mechanisms of pediatric B-ALL, we constructed lncRNA-mRNA co-expression network based on the expression profiles assessing the involvement of lncRNA-mRNA pairs. Our findings not only detect potential lncRNA modules as prognostic biomarkers, but also provide further insight into the molecular mechanisms of action of lncRNAs which may serve as novel therapeutic targets for ALL.

## Materials and methods

### Data accession and preprocessing

The B-ALL transcriptome profiles of 24 ETV6-RUNX1-positive patients and 18 ETV6-RUNX1-negative patients were downloaded from GEO (Gene Expression Omnibus) dataset GSE128254. The expression of 2829 lncRNAs and 1906 mRNAs were measured by LncPathTM Human Cancer Array. The survival analysis was performed using cohort of GDC TARGET-ALL-P3 (https://xenabrowser.net/datapages/), which containing gene expression data and matched survival data. Patients who lacked either the expression data or clinical information were excluded. Finally, we obtained 104 patients for the downstream survival analysis.

### Weighted gene co-expression network analysis

Weighted co-expression network were analyzed by employing R package of WGCNA [25]. One-step method was used for constructing networks and identifying consensus modules. Module Eigengenes (MEs) and phenotypic characteristics of traits were used to evaluate the correlation between the network modules and the positive/negative trait of ALL patients. MEs summarized the expression pattern of modular genes into a single characteristic expression profile. By calculating the correlation coefficient, the module significantly correlated with negative/positive grouping was obtained for subsequent analysis.

### Functional enrichment analysis of module genes

The functional enrichment analysis was conducted on the protein-coding genes of the targeted module by the R package, Clusterprofiler [26]. We used hypergeometric distribution test to identify enriched terms in 3 aspects including biological pathways, molecular functions and cellular components. Pvalues generated by multiple testing were adjusted by False Discovery Rate (FDR) method and the result with adjusted p-value < 0.05 was considered as significant enrichment.

### Construction of lncRNA-mRNA co-expression network

Considering the challenges that lncRNA functions are poorly defined, a co-expression mRNA-based method was generally used to predict the functional mechanisms of lncRNA based on the function of their associated mRNAs [27]. Pearson’s correlation coefficient (PCC) and p-value were calculated for each lncRNA-mRNA pair in the targeted module. The lncRNA-mRNA pairs with an absolute value of the correlation coefficient > 0.6 and p-value < 0.001 were selected to construct lncRNA-mRNA co-expression network (LMCN). The top lncRNA which had the most connections with mRNA were defined as the core of the LMCN.

### Survival analysis

Survival analysis was conducted using survival and survminer package in R. The samples were divided into high group and low group according to the normalized expression value of genes compared with median expression level. Then Kaplan-Meier method was applied to draw survival curves followed by using univariate log-rank test to compare difference between the two groups. P-value <0.05 was considered statistically significant.

## Results

### Weighted gene co-expression network analysis

**Fig 1:**
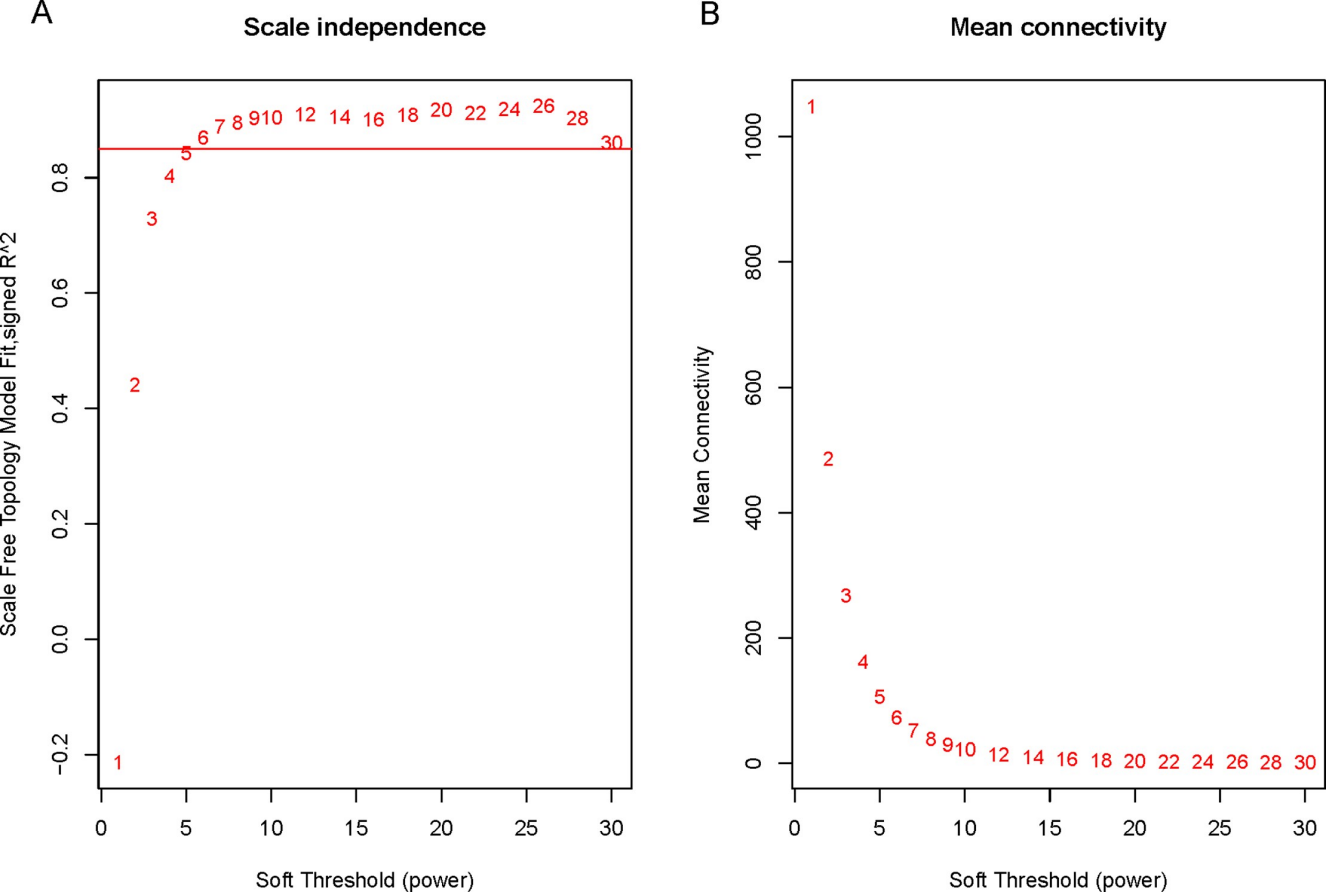
The relationships of Soft Threshold (power) with network properties (scale independence and mean connectivity). (A) Scale Free Topology with the change of power. (B) Mean Connectivity of the network with the change of power. When the power was set to 6, the scale-free topology can exceed 0.85 and the connection was stable. Therefore, we used power = 6 to construct the WGCNA network.

**Fig 2:**
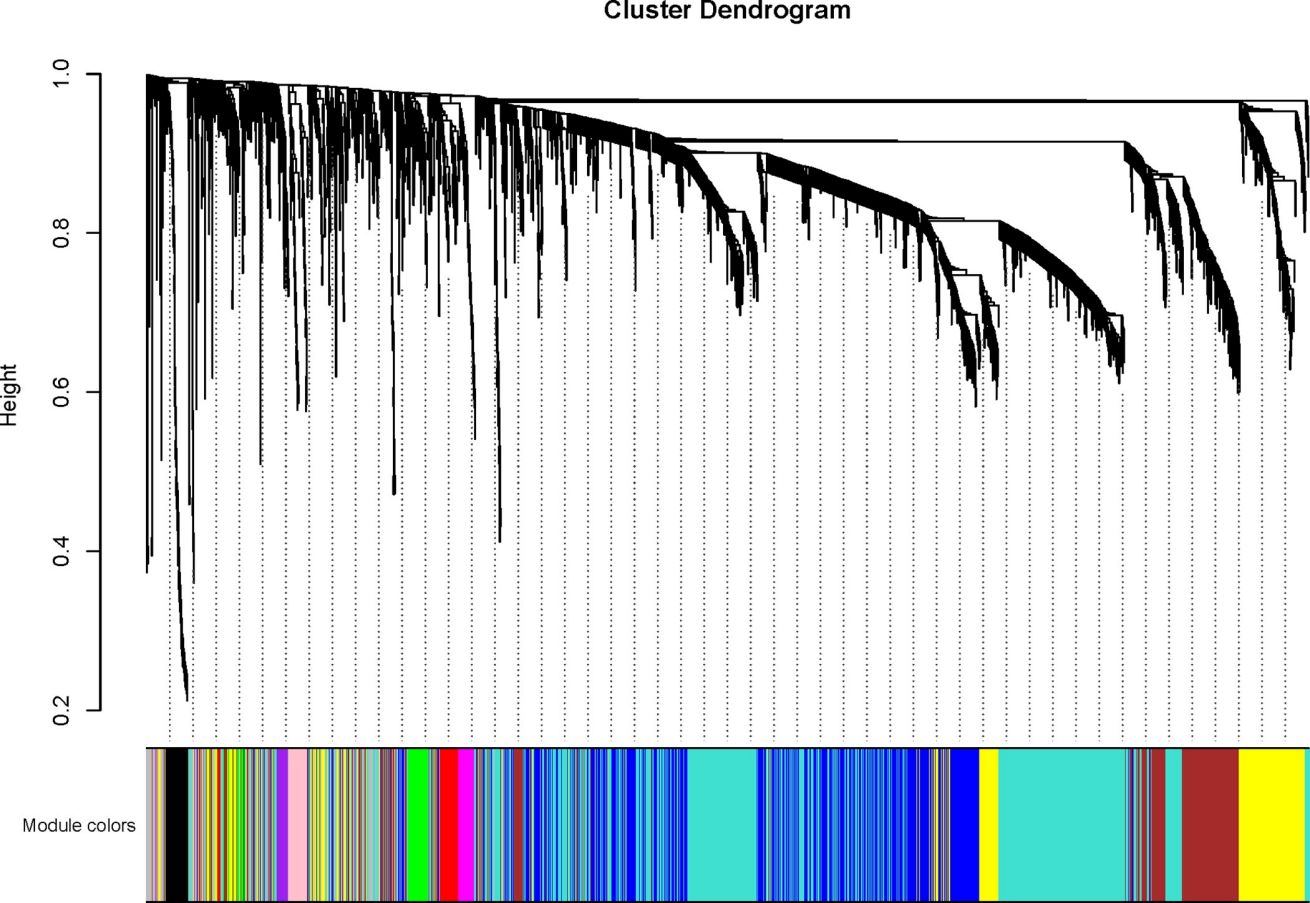
The cluster dendrogram of co-expression network. The genes and lncRNAs were clustered in 11 modules marked with various colors. Members (genes) in same module may participant in common biological process except for grey module which contained unrelated gene members. The largest module was turquoise module which contained 1189 members. And the smallest purple module contained the minimum 43 members.

The expression profile of 41 B-ALL patients and 4735 genes including 2829 lncRNAs and 1906 mRNAs was used to construct co-expression network. The network model fit to be topology scale-free while the thresholding power was set as 6 (Fig 1). Hierarchical clustering divided gene members into 10 modules except the grey module which contained unrelated members (Fig 2). The largest module was turquoise module which contained 1189 gene members. And the smallest purple module contained the minimum 43 members.

### Identification of a module significantly associated with negative/positive trait

**Fig 3:**
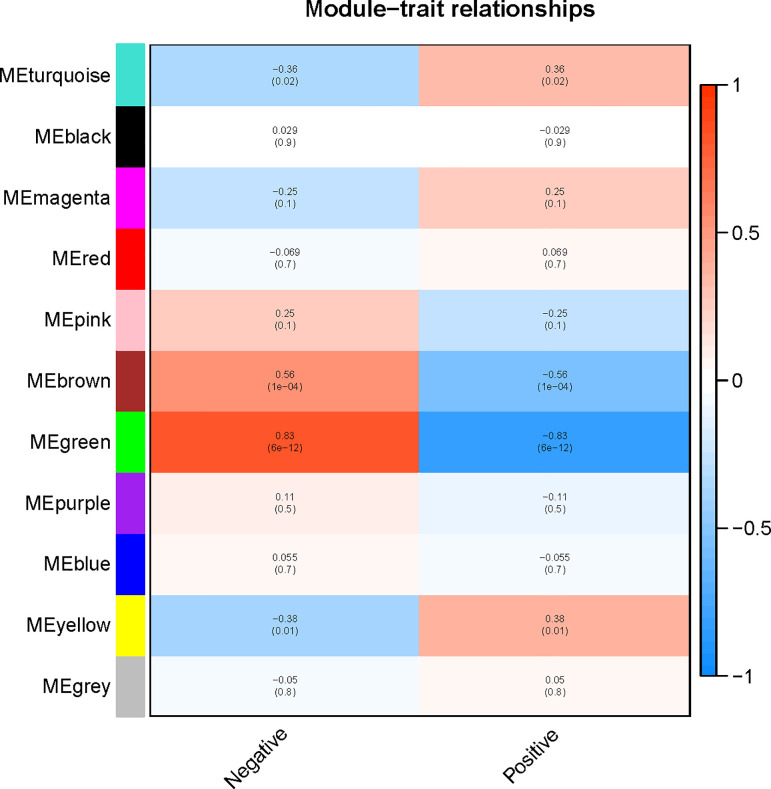
The correlation of each module and positive/negative trait. The green module had a remarkable correlation with the trait. The second significant module was the brown module.

Based on the clinical information, the B-ALL patients were divided into 2 groups: ETV6-RUNX1-positive and ETV6-RUNX1-negative. And we calculated the correlation of each module with positive/negative traits. Among all the modules, we found the green module was significantly correlated with the positive/negative trait (r = 0.83, p = 6 × 10^−12^) (Fig 3). The second significant module was the brown module (r = 0.56, p = 1 × 10^−4^).

### Functional enrichment analysis of modules

**Fig 4:**
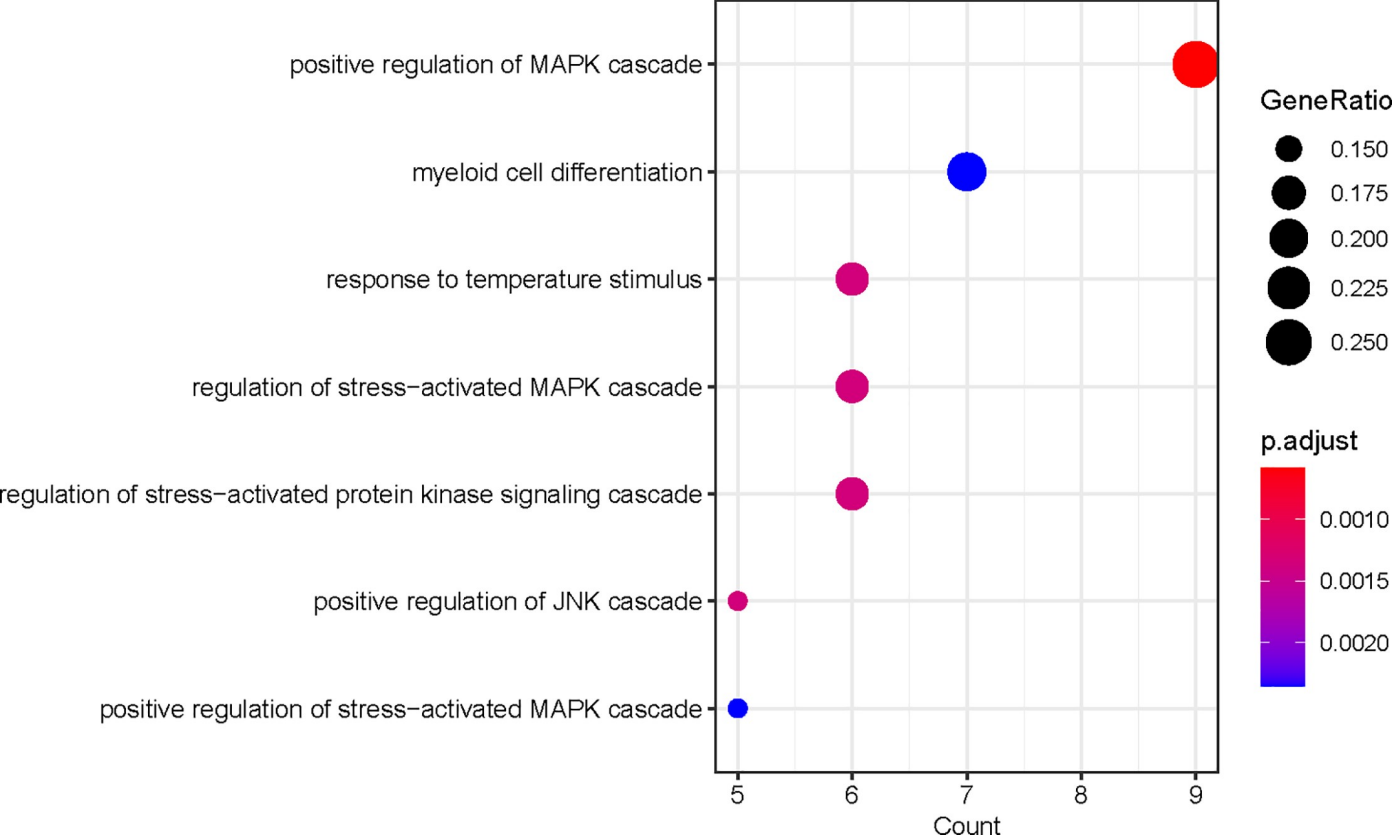
Gene oncology analysis of the genes in the green module. Several signal pathways and leukocyte-related process were enriched.

The gene members belonged to the green module may participate in the same biological process. Therefore, gene oncology analysis was carried out to further investigate the function of them and it showed that the genes were enriched in several terms: positive regulation of MAPK cascade (GO: 0032872), myeloid cell differentiation (GO: 0030099) and positive regulation of JNK cascade (GO: 0046330) (Fig 4). It can be inferred these processes may influence the clinical outcome of B-ALL patients.

### Construction of lncRNA-mRNA co-expression network

**Fig 5:**
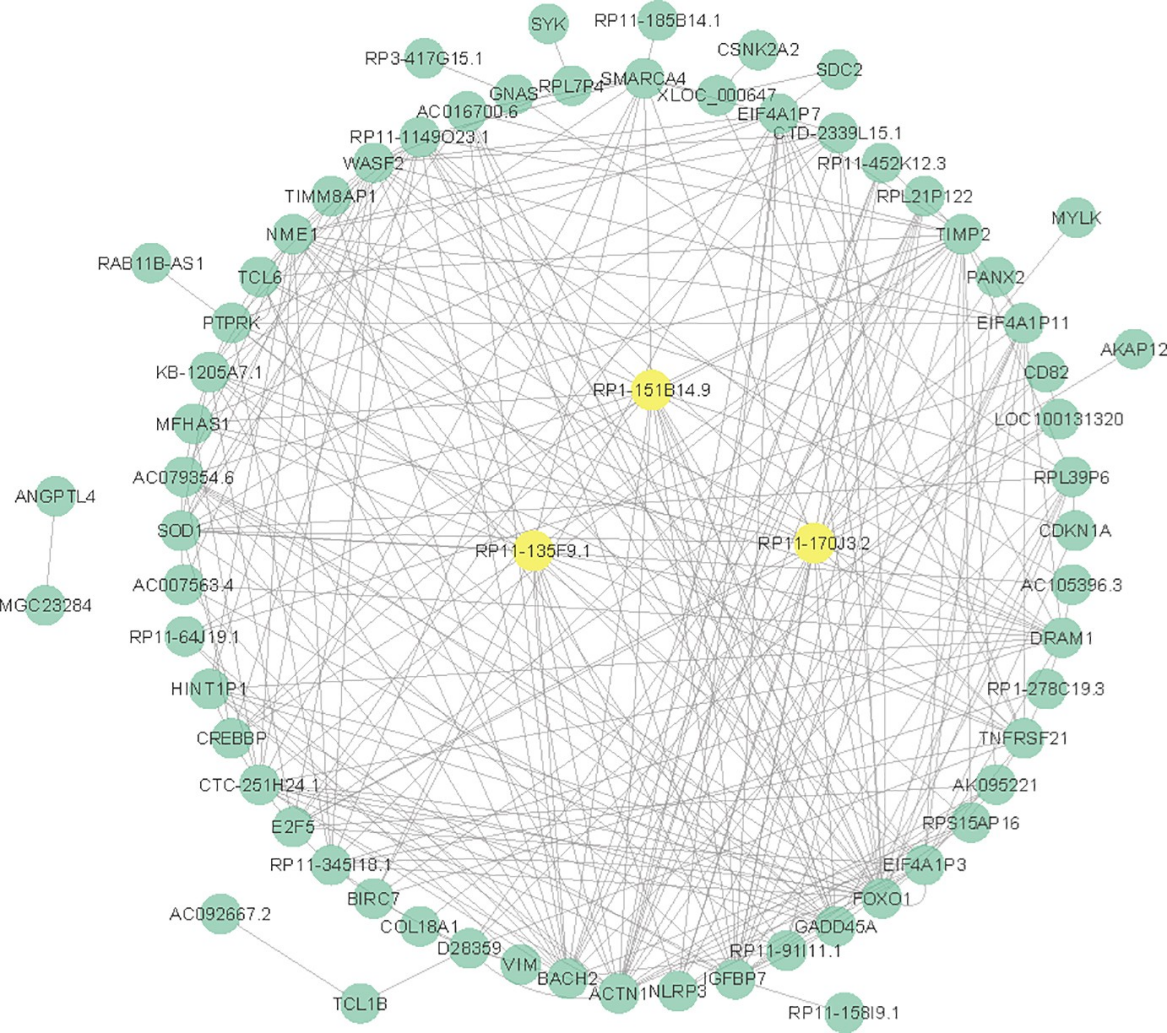
The construction of lncRNA-mRNA network. The lncRNA-mRNA pairs which satisfied PCC >0.6 and p-value <0.001 were shown in the network. RP11-170J3.2, RP11-135F9.1 and RP1-151B14.9 were the top 3 lncRNAs that had the most edges with other mRNA nodes.

We calculated the Pearson correlation coefficient (PCC) of each lncRNA-mRNA pair in green module which included 39 lncRNAs and 44 mRNAs. Then we constructed the lncRNA-mRNA co-expression network (Fig 5). The center of the LMCN was the top 3 lncRNAs (RP11-170J3.2, RP11-135F9.1 and RP1-151B14.9) which had the most connections with mRNAs (data in S1 Table).

### Identification of genes significantly correlated with survival analysis

**Fig 6:**
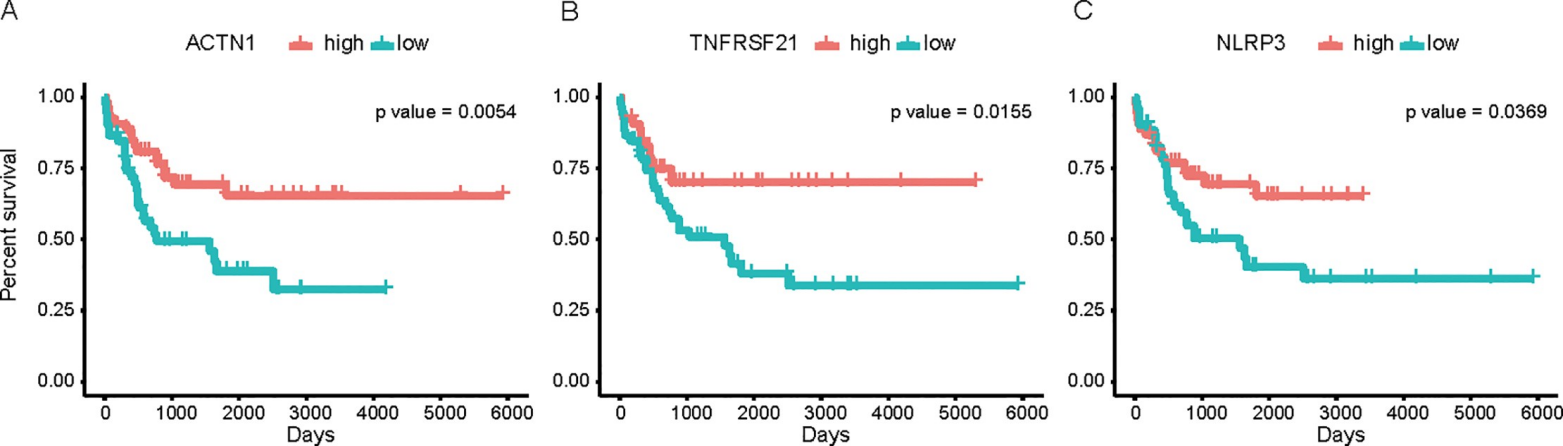
The survival analysis of ACTN1, TNFRSF21 and NLRP3 in patients with ALL. Kaplan-Meier curves for (A) ACTN1, (B) TNFRSF21 and (C) NLRP3. Samples with high expression and low expression of genes were plotted followed by univariate log-rank test. P-values equal to 5.4 × 10^−3^, 1.55 × 10^−2^ and 3.69 × 10^−2^, respectively.

104 ALL patients were separated into high and low group according to the gene expression and p-value was calculated using log-rank test for each gene. Among the mRNAs co-expressed with the top 3 key lncRNAs, we found 3 genes (ACTN1, TNFRSF21 and NLRP3) significantly affected the survival situation of ALL patients and the log-rank p-values were 0.0054, 0.0155 and 0.0369, respectively (Fig 6).

## Discussion

lncRNA is closely related to tumors in the blood system and abnormal expression of lncRNA occurs frequently in leukemia [28]. The diagnose, prognostic and drug resistance function of lncRNA in acute myeloid leukemia (AML) has been widely researched [29–31]. Previous researches have conducted the comprehensive profiles of lncRNAs in pediatric B-ALL by RNA microarray [32,33], while the mRNAs associated with lncRNAs have not been illustrated. This study complements the unexplored area and provides novel clues to molecular mechanism of ALL.

To explore the complex relationships between gene expression profiles and disease traits, a widely applied approach called WGCNA was performed in this study. WGCNA assumes that gene networks obey scale-free distribution. The typical feature of a scale-free network is that most nodes in the network are only connected to few nodes. A fraction of nodes connected with many nodes, which are known as hub or distributed nodes, makes the scale-free network displays a high degree of error tolerance, but extremely vulnerable to collaborative attacks. WGCNA defines the adjacency function formed by gene co-expression correlation matrix and gene network, calculating the dissimilarity coefficients of different nodes, and builds a hierarchical clustering tree based on that. Different branches of the clustering tree represent different gene modules. In Weighted co-expression network analysis, each module represents a group of genes with similar expression profiles. It is similar with cluster analysis, while the difference is that clustering of WGCNA guidelines have biological significance, rather than conventional clustering methods. Therefore, the results obtained by WGCNA method have a higher degree of confidence.

ETV6-RUNX1 gene fusion is frequently detected B-ALL patients. Whether the gene fusion of ETV6-RUNX1 occurred in B-ALL patients leads to different prognosis. The ETV6-RUNX1-positive trait is usually a good prognostic biological marker in pediatric B-ALL. At the same time, it has a greater probability of recurrence in the middle or late stage. However, its internal mechanism about the prognosis and relapse of B-ALL still remains unclear. Here, we identified ten modules including lncRNA and mRNA co-expression relationships from 24 ETV6-RUNX1-positive patients and 18 ETV6-RUNX1-negative patients. After calculating the correlation between modules and the positive/negative traits, we found a gene set (green module) that is the most significantly correlated with gene fusion traits. Functional enrichment analysis revealed genes in green module played a role in several signal pathways including positive regulation of MAPK cascade (GO: 0032872), and positive regulation of JNK cascade (GO: 0046330). These signaling pathways are crucial for regulating genes involved in tumorigenesis including cancer cell migration and proliferation [34]. Previous researches proposed several promising strategy for treatment of acute lymphoblastic leukemia by inhibiting JNK or activating MAPK signaling pathways [35,36]. Besides, the enriched terms included leukocyte-related process including myeloid cell differentiation (GO: 0030099) and regulation of leukocyte differentiation (GO: 1902105). This indicated that the genes in the green module discovered by co-expression network analysis might regulate biological functions in leukemic cells through MAPK and JNK pathways, leading to uncontrolled cell growth and cell cycle regulation.

Consider that both lncRNAs and mRNAs were found in green module, we analyzed the lncRNA-mRNA co-expression relationships to discover the hub molecules in the network and to explore the regulatory correlations. The lncRNA-mRNA pairs which satisfied PCC >0.6 and p-value <0.001 were shown in the network. RP11-170J3.2, RP11-135F9.1 and RP1-151B14.9 were the top three lncRNAs that had the most connections (edges) with other mRNA (nodes). To further investigate the function of these lncRNAs, we paid attention to the mRNAs that they co-expressed with. We found that ACTN1, TNFRSF21 and NLRP3 had a significant correlation with the ALL patient survival prediction. ACTN1 encodes the actin-crosslinking protein α-actinin-1. Actins are the main cytoskeletal proteins that mediate sarcomere function. They also have important non-muscle functions, such as cytokinesis regulation, cell connection and cell migration. Recent studies have confirmed that non-muscular α-actinin is often associated with the occurrence and development of tumors of various tissue types, such as colon cancer, breast cancer, pancreatic cancer, and ovarian cancer [37]. ACTN1 was previously reported to be related with leukocyte development and motility [38]. It is mainly distributed around the microfibrils in non-muscle cells, coupled with actin, connected to the cell membrane and participates in cell adhesion [39]. TNFRSF21 is the member of the recombinant human tumor necrosis factor receptor superfamily. Franiak et al. [40] used fludarabine to induce chronic lymphocytic leukemia (CLL) cells and found TNFRSF21 has a dramatically lower expression compared with control via microarray technology. NLRP3 encodes a pyrin-like protein that is a sensor component of the NLRP3 inflammasome and plays a crucial role in innate immunity and inflammation. NLRP3 was involved in leukemic cell proliferation and death. In lymphocytes derived from chronic lymphocytic leukemia patients, NLRP3 was down-modulated than it from healthy donor [41].

Taken together, we found that the key to progression of ETV6-RUNX1-positive B-ALL is the alteration in biological processes related to the differentiation and growth of leukocytes. The involved mechanism is expression changes of genes in MAPK and JNK signaling pathways mediated by several widely regulated lncRNAs. Three genes play the crucial roles in regulating leukocyte development and proliferation. Accurate detection of these genes expression can help us better predict the prognosis of B-ALL patients, which could serve as promising clinical indicators.

## Conclusion

We integrated weighted gene co-expression network analysis and gene correlation analysis on transcriptome profiles of ETV6-RUNX1-positive and ETV6-RUNX1-negative B-ALL samples. This study discovered crucial lncRNAs (RP11-170J3.2, RP11-135F9.1 and RP1-151B14.9) characterizing two ALL phenotypes, and identified lncRNA-associated genes (ACTN1, TNFRSF21 and NLRP3) that influenced the clinical outcome of patients with ALL. This is the leading research that illustrates the relationship between lncRNAs and acute lymphoblastic leukemia. Our findings provide promising biomarkers for prognosis of patient with acute lymphoblastic leukemia and shed new light on the treatment target of pediatric leukimia.

## Supporting information

S1 Table LncRNAs and corresponding co-expressed mRNAs. (XLSX)
